# The complete chloroplast genome of Mexican marigold (*Tagetes erecta* L., Asteraceae)

**DOI:** 10.1080/23802359.2019.1677191

**Published:** 2019-10-15

**Authors:** Zhi-Cheng Zhang, Shu-Hang Hu, Yong-Qing Peng, Hai-Shan Yan, Fan Xiao, Jing Gao, Jun-Jie Wu, Xuan Zhou, Xin-Yan Xu, Ling Xu, Rui-Hong Wang, Zhe-Chen Qi

**Affiliations:** a Zhejiang Province Key Laboratory of Plant Secondary Metabolism and Regulation, College of Life Sciences and Medicine, Zhejiang Sci-Tech University, Hangzhou, China;; b Shanghai Key Laboratory of Plant Functional Genomics and Resources, Shanghai Chenshan Botanical Garden, Shanghai, China

**Keywords:** *Tagetes erecta*, chloroplast genome, ornamental plant, Mexican marigold

## Abstract

*Tagetes erecta* is an important ornamental and medicinal plant indigenous to Mexico and Guatemala. The complete chloroplast genome of *T. erecta* was newly sequenced in this study. The total chloropalst genome size of *T. erecta* was 152,055 bp. In total, 123 genes were indetified, including 79 protein-coding genes, 8 rRNA genes, and 37 tRNA genes. Twelve genes are containing introns (*ycf3* and *clpP* contained two introns). The overall GC content of this genome was 37.4%. A further phylogenomic analysis of Asteraceae, including 23 taxa, was conducted for the placement of genus Tagetes. The complete plastome of *T. erecta* will provide a valuable resource for further genetic conservation, evolution, and molecular breading studies in Asteraceae.

*Tagetes erecta*, an herb from the Asteraceae family, is native to Mexico and cultivated commercially as a popular garden ornamental for its showy flower head. It is also widely cultivated as a dye plant and source of marigold meal (Tiwary et al. 2014). In addition, it has a wide range of medicinal uses in Americas and Asia. (Negi et al. 2013). The flavonoids, amides, and phenols from *T. erecta* root were reported to have insecticidal and nematicidal effects (Olabiyi and Oyedunmade 2007). Despite its importance in horticulture and medicine, there is little genetic information reported in this genus. Here, we assembled and characterized the plastome of *T. erecta*. It is the first complete chloroplast genome reported in this genus. It would provide potential genetic resources for further evolutionary studies of the genus *Asteraceae* and other relatives.

Total DNA was extracted from fresh leaves of *T. erecta* individual using DNA Plantzol Reagent (Invitrogen, Carlsbad, USA). It is collected from Chun’an, Zhejiang, China (GPS: E118°46’57”, N29°50’45”, Voucher No. ZSTU00821, deposited at Zhejiang Sci-Tech University). The plastome sequences were generated using Illumina HiSeq 2500 platform (Illumina Inc., San Diego, CA, USA). In total, about 14.5 million high-quality clean reads (150 bp PE read length) were generated with adaptors trimmed. Following Liu et al. (2017, 2018), the CLC de novo assembler (CLC Bio, Aarhus, Denmark), BLAST, GeSeq (Tillich et al. 2017), and tRNAscan-SE v1.3.1 (Schattner et al. 2005). were used to align, assemble, and annotate the plastome.

The full length of *T. erecta* chloroplast genome (GenBank Accession No. MN203535) was 152,055 bp and comprised of a large single copy region (LSC with 83,895 bp), a small single copy region (SSC with 18,065 bp), and two inverted repeat regions (IR with 25,048 bp). The overall GC content of the *T. erecta* cp genome was 37.4% and the GC content in the LSC, SSC, and IR regions are 35.4%, 30.9%, and 43.0%, respectively. A total of 123 genes were contained in the cp genome (79 protein-coding genes, 8 rRNA genes, and 37 tRNA genes. Seventeen genes had two copies, which included six PCG genes (*ndhB*, *rpl2*, *rpl23*, *rps7*, *rps19*, and *ycf2*), 7 tRNA genes (*trnA-UGC*, *trnI-GAU*, *trnI-CAU*, *trnL-CAA*, *trnN-GUU*, *trnR-ACG*, and *trnV-GAC*), and all four rRNA species (rrn4.5, rrn5, rrn16, and rrn23). Among the protein-coding genes, two genes (*ycf3* and *clpP*) contained two introns, and other five genes (*ndhA*, *ndhB*, *rpl2*, *rpoC1*, *rps16*,) had one intron each.

Twenty-three chloroplast genome of Asteraceae were fully aligned with MAFFT v7.3 (Katoh and Standley 2013). A best maximum likelihood phylogenetic tree was constructed by IQTREE v1.6.7 (Nguyen et al. 2015), with the K3Pu + F + R3 model and 5000 bootstrap replicates. The result revealed that *T. erecta* belongs to Heliantheae alliance and is sister to a Heliantheae + Madieae + Millerieae + Eupatorieae clade with the current sampling extent (Figure 1).

**Figure 1.**
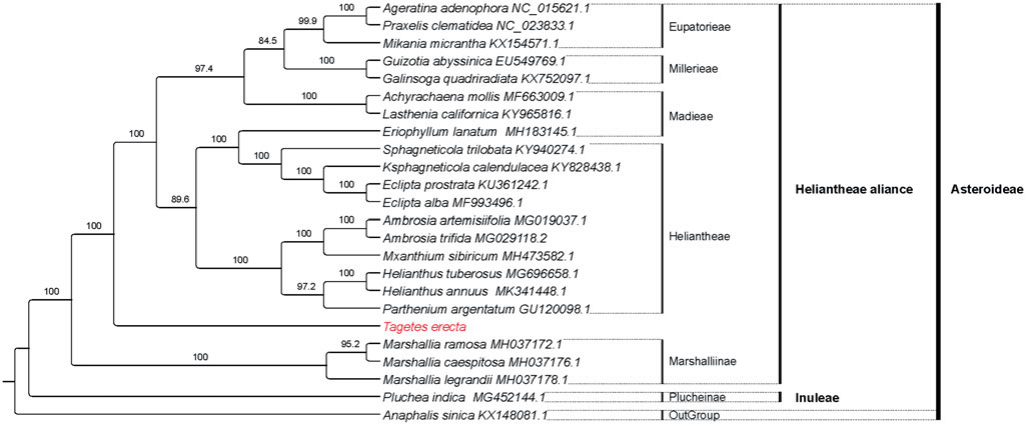
The best maximum likelihood cladogram inferred from 24 chloroplast genomes in Asteroideae (bootstrap values are indicated on the branches).
